# Effectiveness of Chemical and Thermal Treatments on Control *Rhizopus stolonifer* Fruit Infection Comparing Tomato Cultivars with Different Sensitivities to Cracking

**DOI:** 10.3390/ijerph16152754

**Published:** 2019-08-01

**Authors:** Liliana Alfaro-Sifuentes, Melchor Juan, Rosalba Troncoso-Rojas, David Erick Meca, María Antonia Elorrieta, Juan Luis Valenzuela

**Affiliations:** 1 Department of Biology & Geology, Agrifood Campus of International Excellence (CeiA3), and CIEMBITAL, University of Almería, E-04120 Almería, Spain; 2 Laboratory of Phytopathology, Labcolor, Coexphal, Venta el Viso, La Mojonera, E-04746 Almería, Spain; 3 Coordinación de Tecnología de Alimentos de Origen Vegetal, Centro de Investigación en Alimentación y Desarrollo, A. C., Carretera a la Victoria Km. 0.6, Apdo. Postal 1735, Hermosillo, Sonora 83304, Mexico; 4 Estación Experimental Cajamar. Paraje Las Palmerillas 25, El Ejido, 04710 Almería, Spain

**Keywords:** cracking, cultivars, tomato, washing treatments

## Abstract

Tomatoes are among the most important horticultural crops; however, it is estimated that 30% of tomato yield is lost due to postharvest rot due to *Rhizopus stolonifer*, a fungus which requires lesions to initiate the infectious process. Tomato fruit cracking is a physiopathy which causes significant economic losses, since cracking is the door used by the fungus. In this experiment, 14 cultivars of tomato of different types were used. Fruit sampling was carried out in the middle of the crop cycle, coinciding with the peak of yield; then, the fruits were divided into two groups: one group was inoculated with *Rhizopus* in order to assess the effectiveness of washing, whilst the other was treated with sterile water. The fruits of each group were divided into lots to be treated with six washing treatments: dipping in hot water at 20, 40 and 60 °C for 20 s; the fruits were then sprayed with the following solutions: 0.6% of Hydrogen Peroxide 23% + Peracetic acid 15%; commercial bleach at 0.5% and 2% of Hydrogen Peroxide 50%. The control sample was not washed. The results show that there was an influence of cultivar on fruit cracking, which was strongly related with *Rhizopus* infection. Three cultivars were not susceptible to cracking, and therefore, were not sensitive to *Rhizopus* infection. The effectiveness of different washing treatments of tomato fruits depends on several factors; nonetheless, hot water treatment has been shown to be more effective than the use of chemical products such as commercial bleach or hydrogen peroxide. Another factor, the susceptibility of cultivars to cracking, determines the effectiveness of the washing treatment. The results provide an important basis for making decisions about the washing management of tomato fruits in packaging houses.

## 1. Introduction

Tomato (*Solanum lycopersicum*) is one of the most consumed vegetables, and is also the second most important horticultural crop worldwide. The economic and social importance of this crop is reflected in the fact that, worldwide, in 2017, there was a harvested area of 4.8 million hectares and a production of about 183 million tons [1]. It is estimated that more than two million tons of tomatoes for fresh consumption were produced in Spain in 2017 [2], where the province of Almería was the most prolific producer. This province reached a production of nearly 840,000 tons in the 2017–2018 season [3]. However, agricultural products are susceptible attack by microorganisms, causing large losses, mainly due to fungal infections. It is estimated that 30% of tomato crops harvested are lost due to postharvest diseases before reaching the consumer, and about 80% of the total of these losses, both in packaging tomatoes and in bulk, is due to rot caused mainly by *Rhizopus stolonifer* [4]. In the specific case of the South-East Spanish horticultural production, this fungus is the main cause of tomato postharvest losses. *R stolonifer* is a polyphagous saprophytic fungus which can grow over a wide range of temperatures and relative humidities with an optimum temperature of 25 °C. *R. stolonifer* requires lesions caused by insect bites, split or injuries during postharvest management to initiate the infectious process [5,6].Tomato fruit cracking is a physiopathy which causes important economic losses by itself; however, a more significant problem is the fact that the crack is the entry door to fungi, bacteria and pests. In fact, cracking is the main tomato postharvest problem, since it is commonly associated with the growth of *Rhizopus stolonifer*. Although the physiological basis of cracking is not fully understood [7], current studies relate cracking both to cell wall structure and to qualitative and quantitative changes in sugars that could influence water potential [8]. It is very easy to find different degrees of susceptibility to cracking depending on the cultivar, particularly during the postharvest washing process when changes in humidity before and after washing are responsible for cracking [9]. This cracking represents a genuine threat due to microbial spoilage; therefore, postharvest washing tasks become a critical task [10,11]. It is in this context that the susceptibility to cracking of cultivar becomes crucial. Since *R. stolonifer* causes huge post-harvest economic losses, different control methods have been used in the last decade to control it. Methods proposed for this control include, among others: thermal treatments (TT) which include high and low temperatures, chemical treatments (CT), UV-C radiation (UV-C), biological control (BC) and chemical treatments (CT), which include treatments-applying different chemical compounds like chitosan, nitrous oxide, essential oils and other volatile organic compounds [4,12,13,14]. Postharvest heat treatments have been used for the control of fungal diseases in fruits and vegetables. In recent years, these TT’s are interesting from the perspective of reducing the use of postharvest chemical fungicides [15]. Considering that washing is the main step used by the industry during the handling of fresh products, disinfection with chlorinated water is the most common method to disinfect fruits and vegetables, mainly because it is an efficient method, is relatively cheap and can be implemented in operations of any scale [16]. However, there are concerns about its use due to the formation of chlorinated byproducts and therefore, several alternatives, like peracetic acid or hydrogen peroxide, are being used. Peracetic acid is widely used in the processing of fruits and vegetables due to its tolerance to several factors, such as temperature or pH. The products resulting from its decomposition (acetic acid, O_2_, CO_2_ and water) are not harmful to the ecosystem and currently Peracetic acid appears in the federal FDA regulation code as a permitted substance, not exceeding 80 ppm in the washing water [17]. Hydrogen peroxide (H_2_O_2_), which has both bactericidal and fungicidal activity, presents a washing efficiency similar to that of NaClO. Hydrogen peroxide is considered GRAS by the FDA for several food products, although consideration as GRAS for fresh fruit and vegetable is still under consideration [18] However, is a sanitizer used in postharvest processing without risk for human health, when used according the label directions; moreover, risk from dietary exposure through ingestion of fresh fruit and vegetables is not of concern under the current conditions of use [19]. H_2_O_2_ has been shown to inactivate *E. coli* and reduce the population of other pathogens like *Listeria* or numerous fungi in different fruits and vegetables. Moreover, it is also used in freshly-cut products such as cucumbers, lettuces, peppers and melons [16]. On the other hand, the washing task can be done simply with hot water, generally at around 40 °C. In this sense, hot water treatment (HWT) is a technique to control decay in tomatoes. It is considered residue-free and does not require any particular type of regulation [20]. Because HWT implies the immersion of fruits, for tomatoes and other fruits such as pepper, cucumber, citrus or mango, it is most common to use hot water rinse brushing (HWRB), where the fruits are rinsed with tap water and are then sprayed with hot water and forced-air dried while rolling over brushes on a commercial sorting line [21]. This technique is effective in decay control, although many factors can influence decontamination: water temperature and duration of treatment, cultivars, fruit shape and size, crop conditions, maturity degree at harvest, type of pathogen and level of contamination, etc. Taking all these factors into account is therefore essential to the efficacy of the treatment and to achieve a proper rate of heat transfer [15]. However, all these studies have not taken into account the relation between genotype, cracking sensibility, *R. stolonifer* damage and the efficacy of these treatments. Commonly, most of studies look at the control of *R. stolonifer* to avoid fruit rotting but do not consider the fact that cracking (or susceptibility to cracking) facilitates the entry of the fungus into the fruit. It is important to consider that these treatments are surface contact, and it has become necessary to assess different washing treatments, taking into account the different sensitivity to cracking that some cultivars display.

Taking all this into account, the aim of this study was to evaluate, the effectiveness of different chemical treatments (washing fruits with peracetic, hydrogen peroxide and sodium hypochlorite) and thermal treatments (washing at 40 and 60 °C) on different tomato cultivars regarding the control of *R. stolonifer* fruit infection, and to study its relationship to the susceptibility of these different cultivars to fruit cracking.

## 2. Materials and Methods

### 2.1. Plant Material

The plant material used for this study were fruits of 14 cultivars of tomato (*Solanum lycopersicum*) which included different types of tomatoes, as indicated in the Table 1.

The crop was grown in a greenhouse equipped with climate control and automatic fertigation by monitoring the pH and electrical conductivity. All cultivars were grown under coconut fiber with the standard cultural practices for these crops; 84 plots of 20 m^2^ (14 cultivars × 3 repetitions × 2 blocks) with a crop density of 0.8 plant/m^2^ were used in this experiment.

### 2.2. Data Collection

Fruit sampling was carried out in the middle of the crop cycle, coinciding with the peak of yield. The fruits were picked in the commercial ripening stage of maturity, discarding those that were damaged or misshaped, as well as clusters of tomatoes which were in over-mature or immature green stages. After the harvest, the fruits were transported to the Coexphal facilities. In the laboratory, the fruits of each cultivar, repetition and block were divided into two groups. One group was inoculated with a suspension of *Rhizopus stolonifer* at a concentration of 10^6^ CFU per milliliter in order to assess how effective washouts were. The suspension was prepared from a *R. stolonifer* culture in PDA for 6 days in darkness at 24 °C. The other group was treated with sterile water by spraying. Once the applied inoculum or sterile water was dried, the fruits of each group were divided into 7 lots to be treated with the seven following washing treatments: Three physical treatments that consisted of dipping in hot water at 20, 40 and 60 °C for 20 s, denoted as HW20, HW40 and HW60, respectively. The other three chemical treatments consisted of simulating the most common washing method used by packing houses, i.e., spraying the fruits for 5 s with the following solutions: 0.6% of Hydrogen Peroxide 23% + Peracetic acid 15%, Commercial bleach at 0.5% and 2% Hydrogen Peroxide were denoted as HPPA, CB and HP, respectively. A seventh lot was considered as a control and did not receive any washing. For each cultivar group (inoculated or not), washing treatment and control, 3 PET lid containers with 4 or 18 fruits according to tomato type were used. All PET containers were distributed in a randomized block design and stored at room temperature in a dark place. At 3, 6 and 10 days of storage, the incidence of cracking and *Rhizopus* was assessed (presence—1, absence—0).

### 2.3. Statistical Analyses

For statistical analysis, STATGRAPHICS^®^ Centurion XVI (Statpoint Technologies, Inc, Warrenton, VA, USA) was used. The data was treated by ANOVA, followed by a least significant difference test with a significance level of 95%. A Kolmogorov-Smirnov test was used to check the normality of data distribution. When normality failed, the variables were transformed and a non-parametric Kruskal-Wallis test at <0.05 significance level was used.

## 3. Results

### 3.1. Influence of Cultivar on Fruit Cracking and Incidence of Rhizopus Rot in Non-Inoculated Fruits

**Figure 1:**
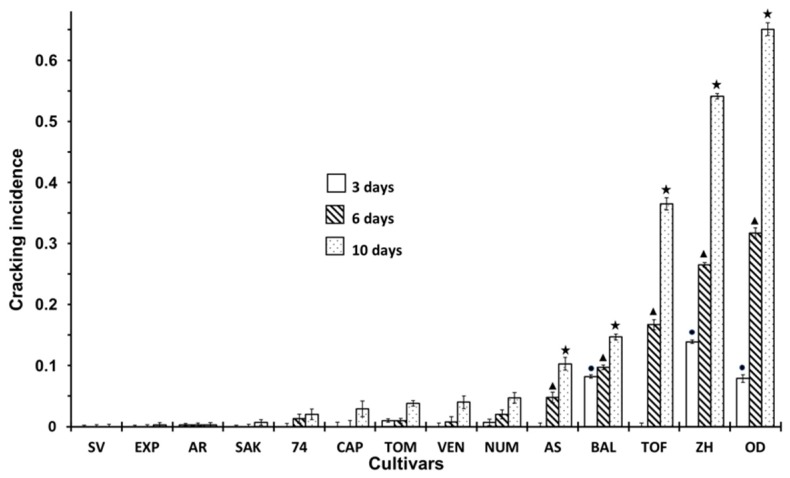
Incidence of Cracking at 3, 6 and 10 days of storage in non-inoculated fruits according to different cultivar. Statistical analysis was performed using Least Significant Difference LSD test at 95%. The star, triangle and circle (*, ●, ▲) indicate significant differences between cultivars on the same day. No symbol in the column indicates no differences.

Figure 1 shows the incidence of fruit cracking in the different cultivars. The mean of cracking incidence denotes the frequency of fruits which showed some degree of cracking, although cracking did not appear in all the varieties. Some of them, such as SV or EXP, showed no signs of this physiopathy, while ZH or OD showed a high incidence of cracking. As shown in Figure 1, 3 cultivars not only showed a higher incidence of cracking, but also an influence of storage time. After 10 days of storage in TOF, ZH and OD, the incidence of cracking was considerably higher than that of another cultivars. In these cultivars, cracking increased until reaching the high value by the end of the experiment. Ten days became critical for TOF, ZH and OD, because it was when large differences appeared compared to previous sampling times, particularly in TOF, which did not show cracking during the 3 first days of storage, but afterwards, showed a significant incidence.

**Figure 2:**
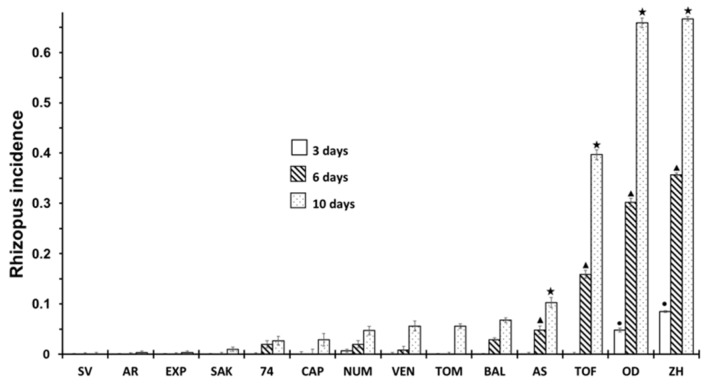
Incidence of *Rhizopus* at 3, 6 and 10 days of storage in non-inoculated fruits according to different cultivar. Statistical analysis was performed using Least Significant Difference LSD test at 95%. The star, triangle and circle (*, ●, ▲) indicate significant differences between cultivars on the same day. No symbol in the column indicates no differences.

The incidence of *R. stolonifer* in the cultivars are shown in Figure 2. There was an increasing incidence of *Rhizopus* as the storage time increased, but the incidence only appeared in a few cultivars; therefore, an influence of cultivars was observed. At three days of storage, the infection was practically non-existence in all cultivars except for OD and ZH, where about 5 and 8% of infected fruits were found. At six days of storage, the AS and TOF cultivars also showed symptoms of infection. After 10 days, the cultivars with a higher incidence of rot were TOF, OD and ZH, while cultivars such as SAK, CAP, VEN, AS or BAL showed a significantly lower incidence of rot, i.e., less than 10%. The most notable results were for SV, EXP and AR, which during the storage time, did not show any sign of rotting. If we compare Figure 1 and Figure 2, we can find that cultivars that have a higher incidence of cracking also present a higher incidence of *Rhizopus* rot.

### 3.2. Influence of Cultivar on Fruit Cracking and Incidence of Rhizopus Rot in Inoculated Fruits

**Figure 3:**
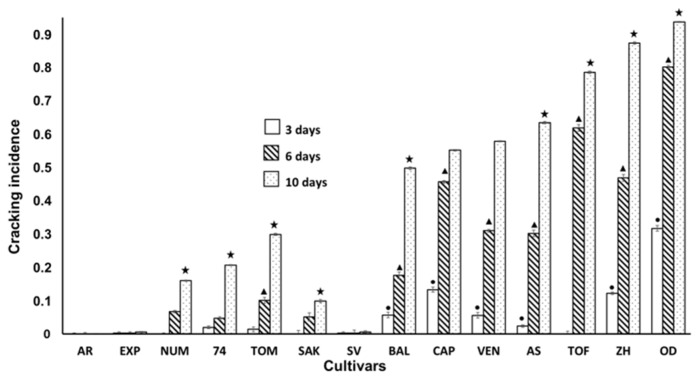
Incidence of Cracking at 3, 6 and 10 days of storage in inoculated fruits according to different cultivar. Statistical analysis was performed using Least Significant Difference LSD test at 95%. The star, triangle and circle (*, ●, ▲) indicate significant differences between cultivars on the same day. No symbol in the column indicates no differences.

Figure 3 shows the incidence of cracking in inoculated fruits. As in the previous analysis performed with non-inoculated fruits, the incidence of cracking increased with the time of storage. In addition, the appearance of cracking was also higher in the same cultivars than in the previous experiment, where the fruits were not inoculated. OD, ZH TOF were the most affected cultivars, although due to inoculation, other cultivars were also affected (Figure 1 and Figure 3); however, the noteworthy result was that cultivars SV, EXP and AR did not show cracking, neither when the fruits were not inoculated, nor when they were. This may indicate that these cultivars can be considered resistant to cracking.

**Figure 4:**
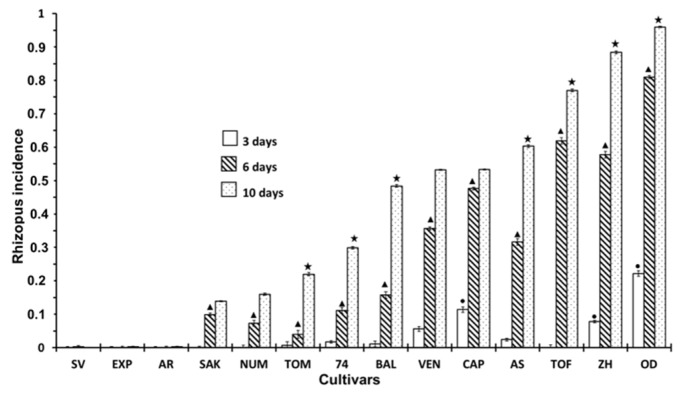
Incidence of *Rhizopus* at 3, 6 and 10 days of storage in inoculated fruits according to different cultivar. Statistical analysis was performed using Least Significant Difference LSD test at 95%. The star, triangle and circle (*, ●, ▲) indicate significant differences between cultivars on the same day. No symbol in the column indicates no differences.

The incidence of *Rhizopus* in inoculated fruits is shown in Figure 4. As expected, the incidence of *Rhizopus* was higher in this case due to artificial inoculation. Most of the cultivars presented severe infections, particularly after 10 days’ storage. Nevertheless, three cultivars, SV, EXP and AR, did not show signs of rot or visual signs of the presence of the fungus, despite being inoculated. Another three cultivars, SAK, NUM and TOM, showed a slight incidence, particularly at 6 days of storage, but did not show signs of infection (or, at least, very light signs) after three days of storage.

### 3.3. Effectiveness of Chemical and Thermal Treatments on the Control of Rhizopus Infection

The evaluation of the different treatments was carried out on fruits that were previously inoculated with *Rhizopus* in order to guarantee the presence of the pathogen, and thus, to be able to evaluate the different treatments. First of all, we present the results of the different washing treatments in a global way. Later, the effects of each treatment according to the cultivar will be discussed.

**Figure 5:**
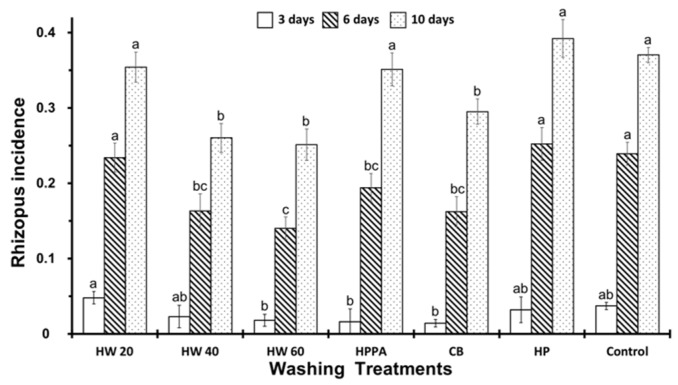
Incidence of Rhizopus at 3, 6, and 10 days of storage in inoculated fruits according to different washing treatments. Washing treatment: HW20, HW40, HW60: Hot water dip at 20, 40, and 60 °C for 20 s; HPPA: spraying 0.6% of Hydrogen Peroxide 23% + peracetic acid 15%; CB spraying Commercial Bleach at 0.5%; HP: spraying 2% of Hydrogen peroxide 50%; control: not washing. On the same day of storage, different letters (a, b, c) indicate significant differences at 5% (LSD test).

As can be seen in Figure 5, the effectiveness of the washing treatments was very similar after three days of storage; only dipping in water at 20 °C showed a slight increase of incidence. Nevertheless, this scenario changed drastically over time; after six days of storage, we observed important differences between treatments. HP and HW20 showed a similar *Rhizopus* incidence to the control, whilst the rest of treatment (HW40, HW60, HPPA and CB) showed a lower *Rhizopus* incidence, particularly HW60, which showed the lowest of all. After 10 days, the incidence of *Rhizopus* was higher than would be expected, although the fruits treated with HW40, HW60 and CB presented a lower incidence.

The results obtained (Table 2) clearly indicate that after 3 days of storage, the incidence of *Rhizopus* is still not detected in most of the cultivars; therefore, we cannot confirm the efficacy of the washing treatments, mainly due to the fact that we could detect *Rhizopus* in several other cultivars without finding differences between washing treatments. Indeed, after three days of storage, a differential effectiveness of different washing treatments could be seen in the ZH and OD cultivars, although not clearly.

After 6 days of storage, the highest incidence appeared in OD, ZH and TOF, followed by CAP, VEN and AS, whilst in three cultivars, SV, EXP and AR, no incidence of *Rhizopus* was detected (Table 3). In relation with the efficacy of washing treatments, the best results were obtained for HW60, followed by HW40 and CB. HPPA and HP showed no differences from the control (non-washed fruits).

Table 4 shows the result for the incidence of *Rhizopus* after 10 days of storage. The incidence took a further turn for the worse, although the behavior of the cultivars and the effectiveness of the washing treatments were similar to what was seen after 6 days of storage. In SV, *Rhizopus* incidence continued persistently undetected, but in EXP and AR, a very slight incidence was noted in the HP and HPPA treatments, respectively, although without statistically significant differences with the control. OD, ZH and TOF continued to show the highest incidences, with most of the washing treatments being statistically equal to the control. HW60 in TOF and ZH was the best option for washing fruits, whilst in OD, it was HPPA. Among the cultivars that did not present incidence of *Rhizopus* and those that had a high incidence, we can find the rest of the cultivars (SAK, NUM, TOM, 74, BAL, VEN, CAP and AS) where, in most cases, the washing treatments showed low efficacy, since the data was statistically similar to the control. However, of particular note is that HW60 was the most effective washing treatment in NUM and 74.

## 4. Discussion

The results were studied from different points of view, firstly, in terms of the relationship between the different varieties and their susceptibility to cracking, both in fruits inoculated with *Rhizopus* and non-inoculated ones. Subsequently, the incidence of *Rhizopus* was evaluated taking the cultivar into account to evaluate the effectiveness of the washing treatments depending on the different cultivars, using fruits inoculated to ensure the presence of the pathogen and, thus, to evaluate the effectiveness of the washing treatments.

A close relationship was found between the incidence of cracking and the incidence of *Rhizopus* in non-inoculated fruits, particularly in some cultivars. These results are not new; it is well established that cultivars which are sensitive to cracking show a higher incidence of *Rhizopus* [5,22]. However, it is also true that infection with *Rhizopus* induces over-ripening of the fruits, causing the emission of volatiles such as ethylene that affect the rest of the fruits which become ripe, thereby favoring cracking [23]. Therefore, it can be considered that these two factors (incidence of cracking and infection with *Rhizopus*) go together. In our study, we have found cultivars that have neither cracking, nor *Rhizopus* infection. This was probably due to their different sensitivities to cracking, rather than not being contaminated with *Rhizopus*. The crop conditions were the same for all cultivars and all samples were handled in the same way during laboratory tests. Therefore, if a contamination occurred in the crop, it would have affected all cultivars equally. In order to verify this point, in the second experiment, the fruits were inoculated and the relationships between cracking and *Rhizopus* infection were observed. Obviously, the incidence of *Rhizopus* was higher, but this confirmed the different susceptibilities to cracking and, consequently, *Rhizopus* infection, since it was found that those varieties which did not crack did not become infected, despite being inoculated.

In relation with washing treatments globally, the results indicated that washing fruits with hot water at 60 or 40 °C was more effective than chemical treatments, probably due to greater capacity of thermal treatments to deepen into the fruit, in contrast to chemical treatments which are contact treatments [13]. In this sense, thermal treatment is able to warm not only the fruit surface but also the underlying layers of the parenchymal tissue through heat transfer. Besides this, all treatments (physical and chemical) have a mode of action, which is to wash off the spores from the fruit epidermis, but thermal treatments have two advantages. On the one hand, the structure of waxes in the cuticle is changed, and this way, micro-cracks or micro wounds are covered by the waxes. This was demonstrated by scanning electron micrographs of cherry tomato fruit epidermis, upon which cracks appeared to be filled with molten wax after the fruits were dipped in hot water 40–45 °C [24]. On the other hand, new anti-fungal compounds are formed after heat treatment [25,26].

Among all of the chemical treatments, CB was the most effective, probably because its residual antimicrobial effect CB promotes a better disinfection, as this residual effect may persist even after three washes, as pointed out in 1998 [27]. This means that CB is a sanitizer with high efficacy at the concentrations assayed [28]. However, peroxide and peracetic acid are very reactive, quickly decomposing to release oxidizing species, and consequently, their residual effect is much lower [29]. Maybe the concentration of these products applied would have to be greater. This notwithstanding, the above evidence, and taking into account the different sensitivity to cracking shown for each cultivar, the effect of washing treatments on each cultivar can be significant. Considering that the fruits were inoculated to verify the effectiveness of the washing treatments, the results clearly indicate that on the one hand there is a varietal influence, and on the other, a different efficacy in the control of *Rhizopus* with the different treatments of washing. The best, in the most of cases, was HW60, regardless of the cultivar. However, HW40 also showed good results without statistical differences with HW60 in some cultivars. These results have been found for other fruits such as pepper, melon, strawberry or citrus [30]. Nonetheless, an important factor which contributes to the efficiency of hot water treatments is the cultivars. In our experiment, we could see how the cultivar factor played a critical role. In this sense, the incidence of *Rhizopus* was almost non-existent during the storage period in three cultivars, whereas in others, no washing treatment was fully effective. This may be due to the different sensitivity to cracking presented by the different cultivars, and thus, those with a zero or low incidence of cracking also showed the best results in the different washing treatments (Figure 2 and Figure 3 and Table 3). Moreover, the postharvest washing task in packaging houses favors cracking when water passes through the epidermis and the stalk of the fruit, increasing the turgor pressure [31]. However, there are genetic differences in the water-absorbing ability by the pericarp, as pointed out in [32], due to differences in the total soluble solids (TSS) between cultivars and the differential presence of other hydrophilic substances, as well as other important factors that depend on genetics, e.g., the thickness of the epidermis, which contributes to cracking resistance [33]. Moreover, changes in the relative humidity and temperature modify the mechanical characteristics of the epidermis [34]. These changes are produced during the washing task, particularly in the packing houses, when the fruits are washed after harvest.

## 5. Conclusions

The effectiveness of different washing treatments of tomato fruits depends on several factors, one being the treatment itself; hot water treatment has been shown to be more effective than chemical products such as commercial bleach or hydrogen peroxide at the concentrations assayed, although this is nuanced, mainly due to the cultivar, since the SV ARS and TR cultivars did not show any infection, and therefore, it would not be more necessary than conventional washing with water to eliminate traces of dust. Others with high cracking sensitivities did not respond to chemical treatment, and maybe it is better to consider heat treatment. Cultivars with medium sensitivities could be exposed to heat and chemical treatments. In the case of HPPA or HP treatment, a greater concentration of the product than that tested here would probably have to be used.

These findings provide an important basis for making decisions in the washing management of tomato fruits in packing houses, due to the different susceptibility to infection by *Rhizopus* according to the sensitivity to cracking. In this sense, some cultivars did not show any infection even after ten days of storage, whilst others showed symptoms of infection early on. Considering that ten days is a usual period of time for fruit packaging, shipping and trading processes, choosing the washing treatment properly, according to cultivar, could be crucial.

## Figures and Tables

**Table 1:** Cultivars and number of fruits used in this experiment.

Type	Cultivar	Number of Fruits
Cocktail vine tomato	TOF	252
	74	300
	BAL	558
	NUM	300
	TOM	576
	OD	252
Cherry plum tomato	AR	672
	SV	672
	SAK	588
	ZH	588
Cherry tomato	EXP	672
Vine tomato	CAP	210
Round tomato	AS	252
Plum tomato	VEN	252

**Table 2:** Incidence of *Rhizopus* on each cultivar at 3 days of storage.

	Physical and Chemical Washing Treatments						
	HW 20	HW 40	HW 60	HPPA	CB	HP	Control
Cultivar							
SV	n.i.	n.i.	n.i.	n.i.	n.i.	n.i.	n.i.
EXP	n.i.	n.i.	n.i.	n.i.	n.i.	n.i.	n.i.
AR	n.i.	n.i.	n.i.	n.i.	n.i.	n.i.	n.i.
SAK	n.i.	n.i.	n.i.	n.i.	n.i.	n.i.	n.i.
NUM	n.i.	n.i.	n.i.	n.i.	n.i.	n.i.	n.i.
TOM	0.06 ± 0.04 a	0.03 ± 0.03 a	n.i.	n.i.	n.i.	n.i.	0.06 ± 0.03 a
74	n.i.	n.i.	n.i.	n.i.	n.i.	n.i.	0.04 ± 0.03 a
BAL	n.i.	n.i.	n.i.	n.i.	n.i.	0.07 ± 0.03 a	n.i.
VEN	0.11 ± 0.05 a	0.06 ± 0.04 a	0.06 ± 0.04 a	n.i.	n.i.	0.11 ± 0.05 a	0.06 ± 0.04 a
CAP	0.13 ± 0.09 a	n.i.	0.13 ± 0.09 a	0.20 ± 0.11 a	0.20 a	n.i.	0.13 ± 0.09 a
AS	n.i.	n.i.	n.i.	n.i.	n.i.	n.i.	0.17 ± 0.06 a
TOF	n.i.	n.i.	n.i.	n.i.	n.i.	n.i.	n.i.
ZH	0.21 ± 0.06 b	0.07 ± 0.04 a	n.i.	0.07 ± 0.04 a	n.i.	0.07 ± 0.06 a	0.10 ± 0.05 a
OD	0.33 ± 0.11 b	0.28 ± 0.11 ab	0.28 ± 0.11 ab	0.06 ± 0.04 a	0.11 ± 0.08 ab	0.33 ± 0.11 b	0.17 ± 0.09 ab

Values of a raw followed by the different letters (a, b, c) mean significant differences at 5% (LSD test). n.i.—no incidence of *R. stolonifer.* Washing treatment: HW20, HW40, HW 60—Hot water dip at 20, 60 and 60 °C for 20 s; HPPA—spraying 0.6% of Hydrogen Peroxide 23% + Peracetic acid 15%; CB—spraying Commercial bleach at 0.5%; HP—spraying x% of Hydrogen Peroxide 50%; Control—no washing.

**Table 3:** Incidence of *Rhizopus* on each cultivar at 6 days of storage.

	Physical and Chemical Washing Treatments						
	HW 20	HW 40	HW 60	HPPA	CB	HP	Control
Cultivar							
SV	n.i.	n.i.	n.i.	n.i.	n.i.	n.i.	n.i.
EXP	n.i.	n.i.	n.i.	n.i.	n.i.	n.i.	n.i.
AR	n.i.	n.i.	n.i.	n.i.	n.i.	n.i.	n.i.
SAK	0.17 ± 0.06 bc	0.10 ± 0.05 ab	0.10 ± 0.05 ab	0.02 ± 0.02 a	0.07 ± 0.04 ab	0.24 ± 0.07 c	n.i.
NUM	0.08 ± 0.06 ab	n.i.	n.i.	0.06 ± 0.06 ab	0.06 ± 0.06 ab	0.17 ± 0.09 b	0.17 ± 0.08 b
TOM	0.08 ± 0.05 a	0.14 ± 0.06 ab	0.06 ± 0.04 abc	0.17 ± 0.05 abc	n.i.	0.04 ± 0.06 c	0.33 ± 0.08 d
74	0.04 ± 0.04 a	n.i.	0.04 ± 0.04 a	n.i.	n.i.	0.11 ± 0.86 a	0.08 ± 0.06 a
BAL	0.08 ± 0.05 ab	n.i.	0.08 ± 0.05 ab	0.22 ± 0.06 bc	0.11 ± 0.05 ab	0.36 ± 0.07 c	0.19 ± 0.07 b
VEN	0.50 ± 0.09 a	0.28 ± 0.08 a	0.22 ± 0.07 a	0.22 ± 0.07 a	0.28 ± 0.07 a	0.50 ± 0.09 a	0.50 ± 0.09 a
CAP	0.53 ± 0.13 a	0.33 ± 0.09 a	0.60 ± 0.11 a	0.53 ± 0.13 a	0.53 ± 0.13 a	0.40 ± 0.08 a	0.40 ± 0.08 a
AS	0.33 ± 0.08 ab	0.11 ± 0.05 a	0.28 ± 0.07 ab	0.33 ± 0.07 ab	0.28 ± 0.78 ab	0.39 ± 0.08 ab	0.50 ± 0.08 b
TOF	0.78 ± 0.07 bc	0.61 ± 0.08 b	0.06 ± 0.04 a	0.56 ± 0.07 b	0.56 ± 0.07 b	0.78 ± 0.06 bc	1,00 ± 0.01 c
ZH	0.79 ± 0.06 c	0.52 ± 0.08 ab	0.40 ± 0.08 a	0.74 ± 0.07 c	0.45 ± 0.08 ab	0.64 ± 0.07 bc	0.50 ± 0.08 ab
OD	0.89 ± 0.08 a	0.94 ± 0.06 a	0.83 ± 0.09 a	0.39 ± 0.12 a	0.83 ± 0.09 a	0.89 ± 0.08 a	0.89 ± 0.08 a

Values of a raw followed by the different letters (a, b, c, d) indicate significant differences at 5% (LSD test). n.i.—no incidence of *R. stolonifer.* Washing treatment: HW20, HW40, HW 60—Hot water dip at 20, 60 and 60 °C for 20 s; HPPA—spraying 0.6% of Hydrogen Peroxide 23% + Peracetic acid 15%; CB—spraying Commercial bleach at 0.5%; HP—spraying x% of Hydrogen Peroxide 50%; Control—no washing.

**Table 4:** Incidence of *Rhizopus* in each cultivar at 10 days of storage.

	Physical and Chemical Washing Treatments						
	HW 20	HW 40	HW 60	HPPA	CB	HP	Control
Cultivar							
SV	n.i.	n.i.	n.i.	n.i.	n.i.	n.i.	n.i.
EXP	n.i.	n.i.	n.i.	n.i.	n.i.	n.i.	n.i.
AR	n.i.	n.i.	n.i.	n.i.	n.i.	n.i.	n.i.
SAK	0.21 ± 0.06 bc	0.12 ± 0.05 ab	0.12 ± 0.05 ab	0.02 ± 0.02 a	0.14 ± 0.05 ab	0.33 ± 0.07 c	0.02 ± 0.02 a
NUM	0.17 ± 0.08 ab	n.i.	n.i.	0.11 ± 0.87 ab	0.17 ± 0.09 ab	0.44 ± 0.12 c	0.29 ± 0.09 bc
TOM	0.17 ± 0.06 a	0.17 ± 0.06 a	0.47 ± 0.08 b	0.44 ± 0.07 b	0.13 ± 0.05 a	0.17 ± 0.05 a	0.61 ± 0.08 b
74	0.33 ± 0.10 bc	0.04 ± 0.06 a	0.17 ± 0.08 ab	0.17 ± 0.09 ab	0.11 ± 0.09 ab	0.17 ± 0.09 ab	0.50 ± 0.10 c
BAL	0.42 ± 0.08 a	0.31 ± 0.08 a	0.22 ± 0.07 a	0.69 ± 0.07 b	0.42 ± 0.07 a	0.82 ± 0.06 b	0.39 ± 0.08 a
VEN	0.61 ± 0.08 a	0.44 ± 0.08 a	0.39 ± 0.08 a	0.56 ± 0.07 a	0.44 ± 0.07 a	0.61 ± 0.07 a	0.67 ± 0.08 a
CAP	0.53 ± 0.13 a	0.53 ± 0.13 a	0.67 ± 0.11 a	0.60 ± 0.13 a	0.53 ± 0.13 a	0.40 ± 0.13 a	0.47 ± 0.11 a
AS	0.89 ± 0.05 d	0.33 ± 0.08 a	0.39 ± 0.08 ab	0.67 ± 0.07 bcd	0.72 ± 0.07 cd	0.72 ± 0.07 cd	0.50 ± 0.08 abc
TOF	0.94 ± 0.04 bc	0.72 ± 0.08 b	0.39 ± 0.08 a	0.78 ± 0.07 bc	0.72 ± 0.07 b	0.83 ± 0.06 bc	1.00 ± 0.01 c
ZH	1.00 ± 0.01 c	0.88 ± 0.05 bc	0.62 ± 0.08 a	0.93 ± 0.04 bc	0.83 ± 0.06 b	0.95 ± 0.03 bc	0.98 ± 0.02 c
OD	1.00 ± 0.01 b	1.00 ± 0.01 b	1.00 ± 0.01 b	0.72 ± 0.01 a	1.00± 0.01 b	1.00 ± 0.01 b	1.00 ± 0.01 b

Values of a raw followed by the different letters (a, b, c, d) mean significant differences at 5% (LSD test). n.i.: no incidence of *R. stolonifera;* Washing treatment: HW20, HW40, HW 60: Hot water dip at 20, 60 and 60 °C for 20 s; HPPA: spraying 0.6% of Hydrogen Peroxide 23% + Peracetic acid 15%; CB: spraying Commercial bleach at 0.5%; HP: spraying x% of Hydrogen Peroxide 50%; Control: no washing.
